# Effect of CPPU on Carbohydrate and Endogenous Hormone Levels in Young Macadamia Fruit

**DOI:** 10.1371/journal.pone.0158705

**Published:** 2016-07-07

**Authors:** Hui Zeng, Weihai Yang, Chaozhong Lu, Wenqiu Lin, Minghong Zou, Hanzhou Zhang, Jifeng Wan, Xuming Huang

**Affiliations:** 1 College of Horticulture, South China Agricultural University, Guangzhou, Guangdong, China; 2 South Subtropical Crop Research Institute, Chinese Academy of Tropical Agricultural Sciences, Zhanjiang, Guangdong, China; 3 Key Laboratory of Tropical Fruit Biology, Ministry of Agriculture, Zhanjiang, Guangdong, China; Beijing Forestry University, CHINA

## Abstract

*N*-(2-Chloro-4-pyridyl)-*N′*-phenylurea (CPPU) is a highly active cytokinin-like plant growth regulator that promotes chlorophyll biosynthesis, cell division, and cell expansion. It also increases fruit set and accelerates fruit enlargement. However, there has been no report about the effect of CPPU on fruit development and its physiological mechanism in macadamia. In this study, we investigated the effect of CPPU treatment at early fruit development via foliar spray or raceme soaking at 20 mg·L^-1^ on fruit set and related physiology in macadamia. Changes in carbohydrate contents and endogenous hormones in leaves, bearing shoots and fruit were also examined. Results showed that CPPU significantly reduced young fruit drop and delayed the wave of fruit drop by 1–2 weeks. The treatment significantly decreased the contents of total soluble sugars and starch in the leaves, but increased them in the bearing shoots and total soluble sugars in the husk (pericarp) and seeds. These findings suggested that CPPU promoted carbohydrate mobilization from the leaves to the fruit. In addition, CPPU increased the contents of indole-3-acetic acid (IAA), gibberellin acid (GA_3_), and zeatin riboside (ZR) and decreased the abscisic acid (ABA) in the husk. Therefore, CPPU treatment reduced the early fruit drop by increasing carbohydrate availability and by modifying the balance among endogenous hormones.

## Introduction

The cultivated macadamias (*Macadamia integrifolia* Maiden and Betche, *M*. *tetraphylla* Johnson, and hybrids) are evergreen trees originated in the eastern Australia and grown commercially in subtropical regions across the world for their nutritious kernel. In China, macadamia was first introduced and planted experimentally in the late 1970s, and an industry of about 80,000 ha has developed in the subsequent 35 years, accounting for more than half of the world’s planting area [1]. However, production of macadamia in China was very low due to low tree productivity even in the mature orchards [1]. The total production of the nut in shell in China was only about 9,700 tonnes in 2013.

Macadamia trees usually produce numerous flowers borne on axillary racemes, and a mature tree can produce more than 10,000 racemes each consisting of 100–300 flowers [2]. However, less than 10% of flowers can be successfully fertilized and set young fruit in 2 weeks after anthesis [3, 4], and 80% of the premature fruit are abscised in the following 8 weeks, which is presumably caused by a shortage of carbohydrates for rapid fruit development [4–8]. The excessive fruit drop is a common problem in macadamia orchards across the production regions in China, including Guangdong [9], Yunnan [10], and Guangxi [11]. Thus, this phenomenon has posed a major challenge to the development of the macadamia industry.

Plant growth regulators are applied to manipulate premature fruit drop for increasing fruit set or fruit thinning [7, 12, 13]. The effect of plant growth regulators in the reduction of premature fruit drop in macadamia has been assessed. It has been demonstrated that premature fruit drop in macadamia could be inhibited by 1-naphthaleneacetic acid (NAA) and 2,4-dichlorophenoxyacetic acid in detached racemes collected 4–6 weeks after anthesis [4], but no significant increase in initial fruit set was observed after NAA treatment in on-tree racemes [14, 15]. When benzyladenine (BA) [2] or aminoethoxyyinylglycine (an ethylene biosynthesis inhibitor) [16] was applied to racemes before or after anthesis, fruit set per raceme in the orchard was slightly increased within 8–10 weeks after anthesis. Studies also demonstrated that GA_3_ and paclobutrazol were ineffective in increasing the final fruit set [2, 4].

CPPU is a highly active synthetic cytokinin that promotes chlorophyll biosynthesis, cell division, and cell expansion [17, 18]. This compound also increases fruit set and accelerates fruit enlargement, and has been tested extensively in fruit crops including apple [12], blueberry [19, 20], citrus [21], Kiwifruit [17], persimmon [22] and pear [23]. There is evidence that CPPU promotes fruit development in kiwifruit by increasing endogenous cytokinins, gibberellins and auxins [24, 25], which results in a greater sink strength in the fruit [26, 27]. The application of CPPU together with foliar fertilizer also significantly increases fruit set and yield in macadamia [28]. However, there has been no report about the effect of CPPU treatment alone on fruit retention in macadamia. In this study, we assessed the effect of CPPU treatment with foliar spray or raceme soaking on fruit abscission during early fruit development, and discussed its influences on carbohydrate availability and endogenous hormones.

## Materials and Methods

### Plant Materials

Our experiment was performed in a mature macadamia orchard located at South Subtropical Crops Research Institute (110.3°E,21.2°N), Zhanjiang, China, during March and July, 2014. Trees in the orchard were grown at 5 by 6 meters. Six 9-year-old trees of ‘Nanya-2’ (*M*. *integrifolia*) with similar canopy sizes and initial fruit load were chosen for the experiments, which were carried out with 3 replicates (n = 3) using individual trees as the experimental plot or block. The CPPU reagent was purchased from Sangon Biotech (Shanghai, China).

### Foliar spray of CPPU

The experiment was carried out randomized design with 3 replicates using individual trees as the experimental plot. CPPU solution at 20 mg·L^-1^ was sprayed onto the canopy of the selected trees at 15 days after anthesis, and another three trees were sprayed with water and used as control. Sixty similar sized racemes with comparable initial fruit set at different positions of the canopy were selected from each tree and then marked for observation, and the number of fruit on each marked raceme was recorded. The mature leaves at the second and third nodes on the bearing shoots and the stems of these bearing shoots were collected at regular intervals. The samples were dried and ground into fine powders for later analysis.

### Raceme soaking with CPPU

The experiment was carried out using randomized block design with 3 replicates using three trees as the experimental blocks. Two hundred similar sized racemes with similar initial fruit set were marked at 17 days after anthesis from different canopy positions of each tree. 100 of them were soaked in CPPU solution (20 mg·L^-1^) for 1 min, and the remaining 100 racemes were soaked with water and used as the control. For each treatment, 50 of the selected racemes were used for recording the number of fruit per raceme, and the other 50 for fruit sampling. The sampled fruit from each treatment were separated into the husk and seed, and rapidly frozen and ground to fine powder in liquid nitrogen and stored at -80°C for lab analyses.

### Investigation of fruit set

After CPPU treatment, the number of fruit on each marked raceme was recorded, and the average number of fruit per raceme, accumulative fruit drop rate, and relative fruit drop rate were determined. The average number of fruit per raceme was calculated as the mean fruit number of all marked racemes, the accumulative fruit drop rate was the percentage of the total number of fruit drop from the day of treatment against the initial fruit set. The relative fruit drop rate was the percentage of fruit drop during the period between two investigation dates against the fruit number per raceme on the date of the first investigation of the two.

### Measurement of total soluble sugars

Soluble sugars were extracted from the powdered samples (0.5 g) as described by Yang et al. [29] with some modification. Briefly, each sample was homogenized with 10 ml of 80% ethanol and then water bathed at 85°C for 1 h. The mixture was centrifuged at 8000 × *g* for 20 min at 4°C, and the precipitate was extracted again with 10 ml 80% ethanol and the above mentioned procedures were repeated. The supernatants of the two centrifuges were mixed and adjusted to 20 ml, to which active carbon was added to remove chromatic substances. After being filtered, 10 ml filtrate was evaporated to remove ethanol in 85°C water bath, and the condensate was diluted to a volume of 5 ml with distilled water. The anthrone method was performed to measure the total soluble sugars in the extract using sucrose as the standard [30].

### Measurement of the starch content

The sample (0.2 g) was homogenized with 10 ml of 80% ethanol and water bathed at 85°C for 1 h. The mixture was centrifuged at 8000 × *g* for 15 min and the supernatant was discarded. After repeating the above-mentioned procedures, the precipitate was re-suspended in 10 ml 80% (w/v) calcium nitrate, and placed in a boiling water bath for 1 h. After filtration. the filtrate was collected and used for the measurement of starch content using the I_2_–KI method [31].

### Measurement of carbohydrate composition

Carbohydrates were extracted and determined according to the protocol of Hu et al. [32] with modifications. Sample (0.5 g) was homogenized with 5 ml 80% ethanol and water bathed at 85°C for 20 min. After centrifuge at 8000 × g for 20 min at 4°C, the precipitate was extracted again with 5 ml 80% ethanol following the above-mentioned procedure. The supernatants of the two centrifuges were combined, adjusted to 10 ml, and evaporated in 85°C water bath to remove ethanol. The condensate was diluted to a volume of 2 ml with distilled water and filtered. The filtrate was used for high-performance liquid chromatography (HPLC) analysis using 75% acetonitrile as the mobile phase, which had been ultrasonically degassed for 2 h before use. A sample volume of 10 μL, a flow speed of 1.0 mL/min and a column temperature of 35°C were applied. The HPLC system (Shimadzu, Japan) used was with an Agilent NH_2_ chromatographic column (150 mm × 4.6 mm) and a refractive index detector. The quantification of carbohydrates was performed according to external standard solution calibration. Standards sugars (sucrose, fructose, and glucose) were purchased from Sigma Chemical Co.

### Measurement of endogenous hormones

The extraction, purification and determination of endogenous levels of IAA, GA_3_, ZR and ABA were performed using a modified method of enzyme-linked immune sorbent assay (ELISA) described by He [33], Wu et al. [34] and Yang et al. [35]. Sample (0.5 g) was homogenized in a mortar (at 0°C) with a small amount of PVPP and 5 ml phosphate buffer solution (PBS) (50 mmol/L, pH 7.5) containing 80% methanol and 1 mM butylated hydroxytoluence. The homogenate was centrifuged at 8000 × g for 20 min at 4°C, and the supernatant was forced to pass through a C_18_ Sep-Pak cartridge (Waters Corp., Millford, MA, USA), which was prewashed successively with 10 mL 100% and 5 mL 80% methanol. The hormone fractions were dried by N_2_ blowing and dissolved in 2 mL (50 mmol/L, pH 7.5) for hormone analysis by ELISA.

The mouse monoclonal antigen and antibodies against IAA, GA_3_, ZR and ABA, and immunoglobulin G-horse radish peroxidase (IgG-HRP) used in ELISA assays were produced by the Phytohormones Research Institute, China Agricultural University, China [33]. The quantification of these endogenous hormones was performed using standard curves, which were all generated at high coefficients of quadratic correlation (R^2^>0.998).

### Statistical analysis

Differences between the treatment the control were analyzed using the procedure of independent sample T Test performed by SPSS statistical analysis software (Version 11.5.0, SPSS Inc.).

## Results

### Effect of CPPU on young fruit drop

**Fig 1:**
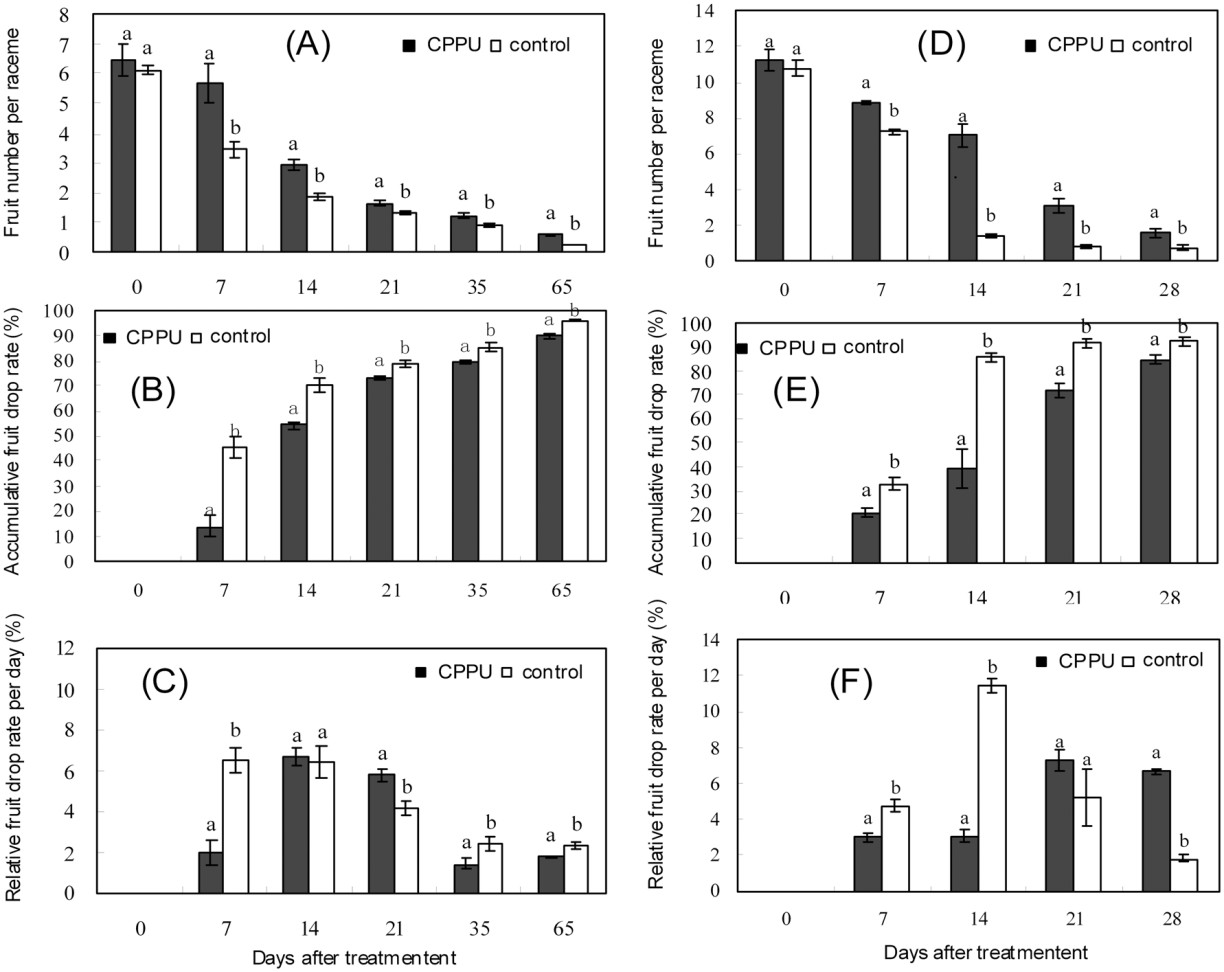
Effect of CPPU treatments, foliar spraying (A, B and C), and CPPU fruit raceme soaking (D, E, and F) on early fruit drop in macadamia. Different letters indicate significant difference between the treatment and the control based on *t*-test (*p*<0.05).

Fruit set per raceme was significantly increased by foliar spray of CPPU at 15 days after anthesis, and the average number of fruit per raceme was 0.57 at 65 days after treatment (80 days after anthesis), which was 2.4 folds greater than the control (Fig 1A). Within the 21 days after CPPU treatment (15 to 36 days after anthesis), the accumulative fruit drop rate in the treated trees increased rapidly to 73.3%, which was significantly lower than 79.0% in the control. From 21 to 65 days after treatment, the accumulative fruit drop rate in the treatment maintained significantly lower than that in the control, but the increase in cumulative fruit drop rate during this period was comparable between the treatment and the control, which was 16.9% and 17.2%, respectively (Fig 1B). These results suggest that fruit drop mainly occurred during early fruit development. During the first week after CPPU treatment, the relative fruit drop rate in the treatment was 13.8%, which was significantly lower than 45.5% in the control (Fig 1C). Then, it increased rapidly to a peak value of 46.7% during the second week after treatment, which was comparable to that in the control (44.7%). Subsequently, the relative fruit drop rate gradually reduced from the second to the fifth week after the treatment, and the rate was about half that of the control in the fifth week. It increased again to 53.3% from 35 days to 65 days after treatment, which was significantly lower than 70.0% in the control, indicating that foliar application of CPPU was effective in suppressing fruit abscission and delayed the occurrence of fruit drop peak by about 1 week in macadamia.

In the case of raceme soaking treatment with CPPU solution at 17 days after anthesis, the average fruit number per raceme was 1.54 in the fourth week after treatment, which was about 2 folds higher than that of the control (Fig 1D). The accumulative fruit drop rate increased to 84.6% in the treatment within four weeks (Fig 1E) compared to 92.2% in the control. The relative fruit drop rate in the treated racemes was significantly lower than that in the control within the first two weeks after treatment, but an opposite pattern was found in the following two weeks (Fig 1F). Relative fruit drop rate peaked at the second week after treatment in the control but at the third week in the treatment, and the peak value of the control was significantly higher than that of the treatment. The results indicated that raceme soaking with CPPU reduced fruit drop and delayed the peak of young fruit drop.

In summary, both foliar spray and raceme soaking with CPPU positively affected fruit retention in macadamia during early fruit development. The application of CPPU not only reduced early fruit drop but also delayed the abscission process. However, raceme soaking with CPPU was more effective than foliar spray.

### Effect of foliar application of CPPU on carbohydrate contents in leaves and bearing shoots

**Fig 2:**
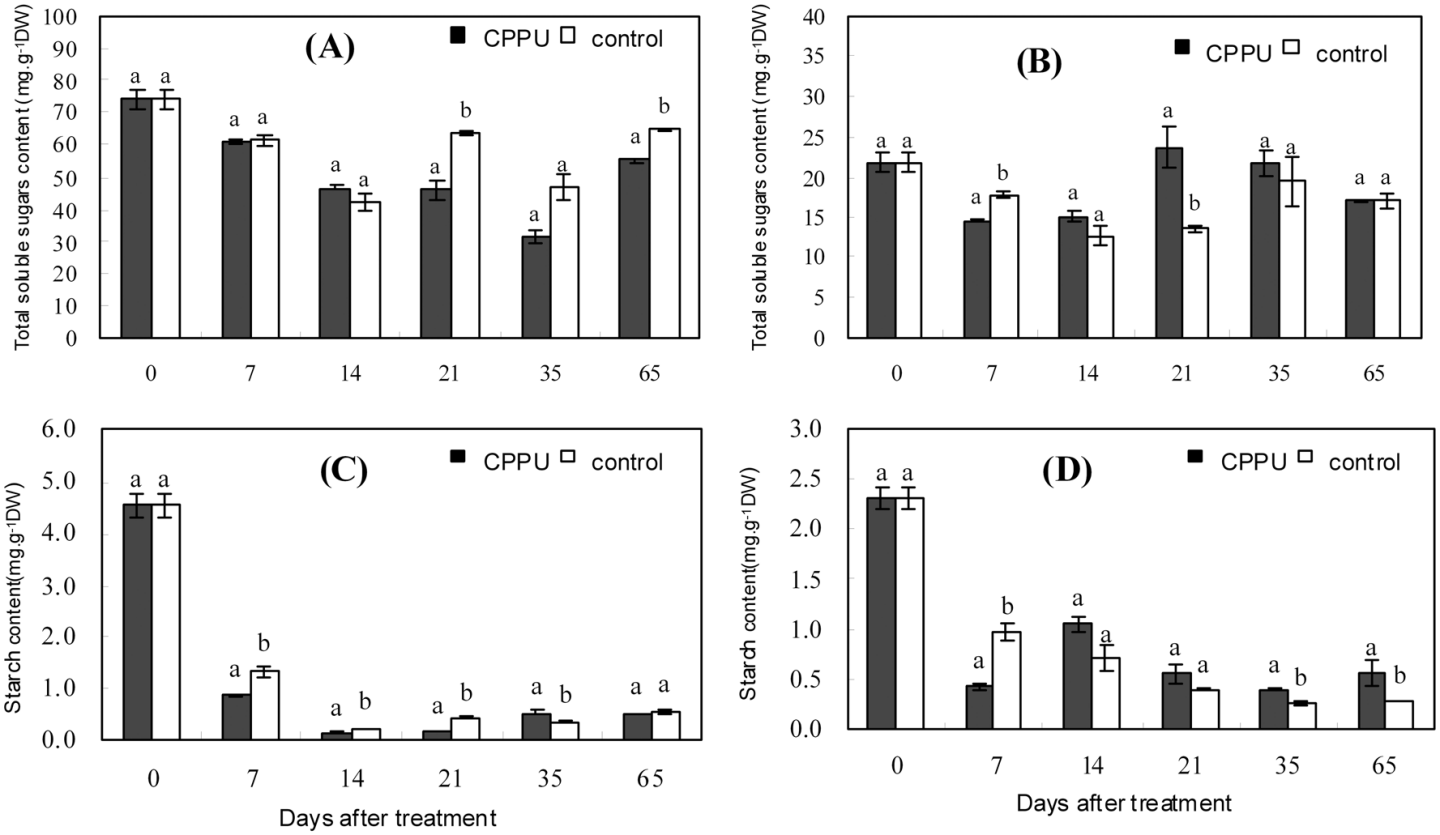
Effect of foliar spray of CPPU on the carbohydrate contents in leaves (A and C) and bearing shoots (B and D) in macadamia. Different letters indicate significant difference between the treatment and the control based on *t*-test (*p*<0.05).

The change patterns of total soluble sugars (Fig 2A) and starch (Fig 2C) in the leaves were similar in the control and the treatment, decreasing initially and increasing later. However, the levels of total soluble sugars and starch in the treatment were significantly lower than those in the control in the period from 21 to 65 days and from 7 to 21 days after treatment, respectively. The results indicated that foliar spray of CPPU might accelerate the utilization and export of assimilates from the leaves and thus decrease the carbohydrate level in the leaves.

The change in total soluble sugars in the bearing shoots of the treated trees showed a similar pattern to that in the control, but the peak in the treated bearing shoots occurred at 21 days after treatment, which was two weeks earlier than that in the control (Fig 2B). In addition, the level of total soluble sugars in the treatment was significantly lower than that in the control at 7 days after treatment but it increased and became significantly higher than that in the control at 21 day after treatment. Starch content in the bearing shoots displayed a declining trend in both the treatment and the control (Fig 2D). However, CPPU treatment significantly reduced starch content at 7 days after treatment, but increased it at day 35 and day 65. The results indicated that foliar spray of CPPU promoted the accumulation of carbon nutrition in the bearing shoots.

### Effect of raceme soaking with CPPU on the carbohydrates in fruit tissues

**Fig 3:**
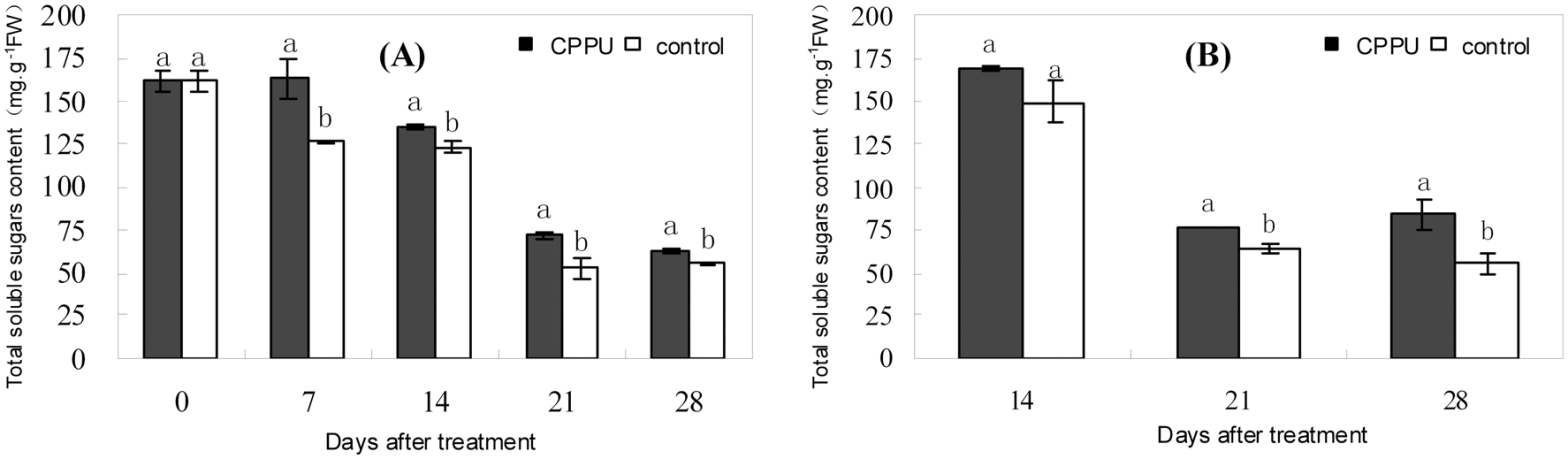
Effect of raceme soaking with CPPU on the total soluble sugar content in the husk (A) and seed (B). Different letters indicate significant difference between the treatment and the control based on *t*-test (*p*<0.05).

**Fig 4:**
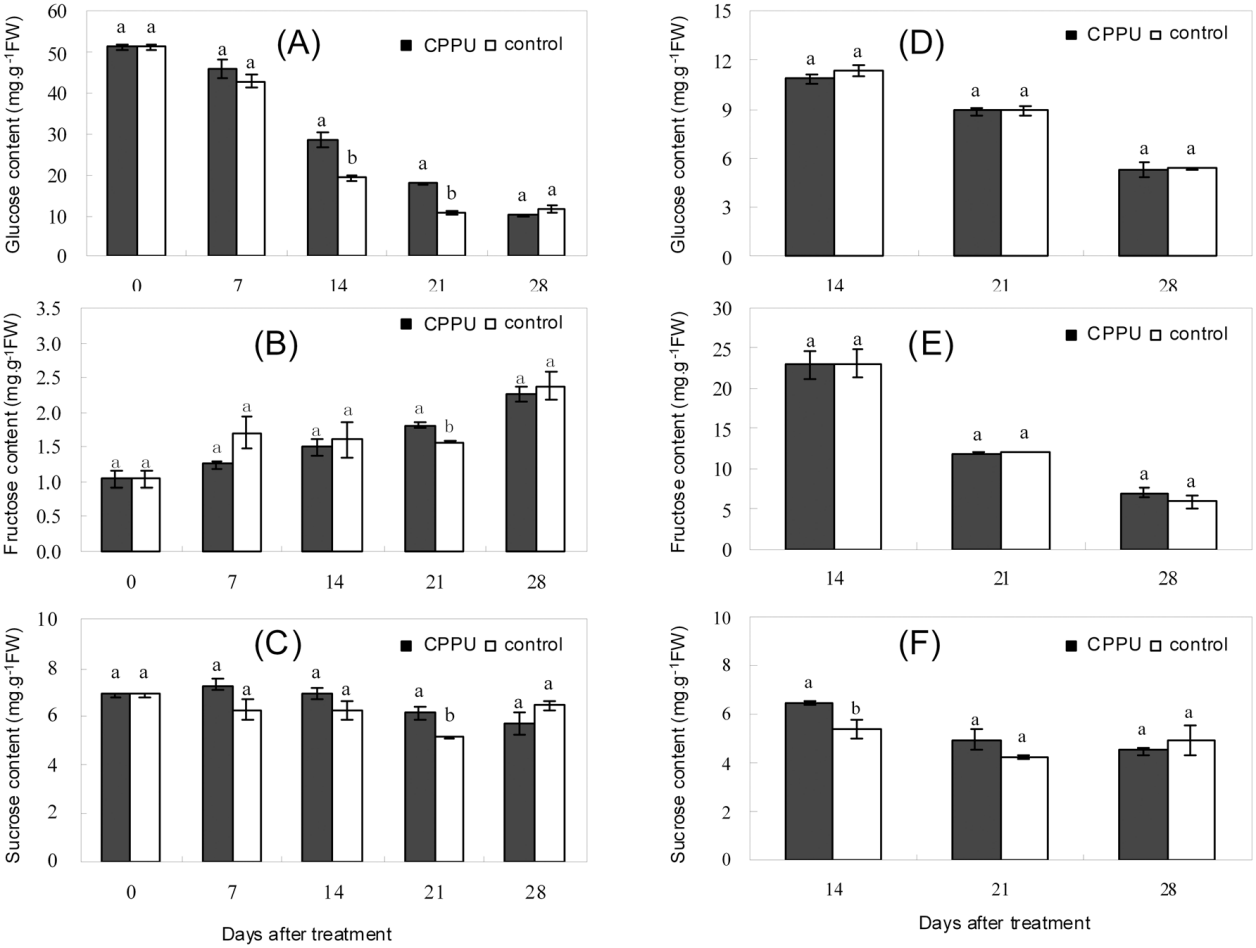
Effect of raceme soaking with CPPU on carbohydrate composition in the husk (A, B, and C) and seed (D, E, and F). Different letters indicate significant difference between the treatment and the control based on *t*-test (*p*<0.05).

The total soluble sugar content in the husk and seed gradually decreased during the early fruit development, and CPPU soaking significantly increased the content of total soluble sugars (Fig 3). The levels of various sugars also decreased in both the husk and the seed with the development of fruit, except for fructose in the husk, which increased steadily (Fig 4). Glucose, fructose, and sucrose contents in the husk were significantly increased by CPPU soaking compared with the control at day 21 after treatment (Fig 4A, 4B and 4C). The sugar contents in the seed were generally not influenced by CPPU treatment except for sucrose, which was significantly increased by CPPU at day 14 (Fig 4D, 4E and 4F). The results suggested that raceme soaking with CPPU exerted a greater effect on the sugar composition in the husk than in the seed.

### Effect of raceme soaking with CPPU on the endogenous hormones in the fruit

**Fig 5:**
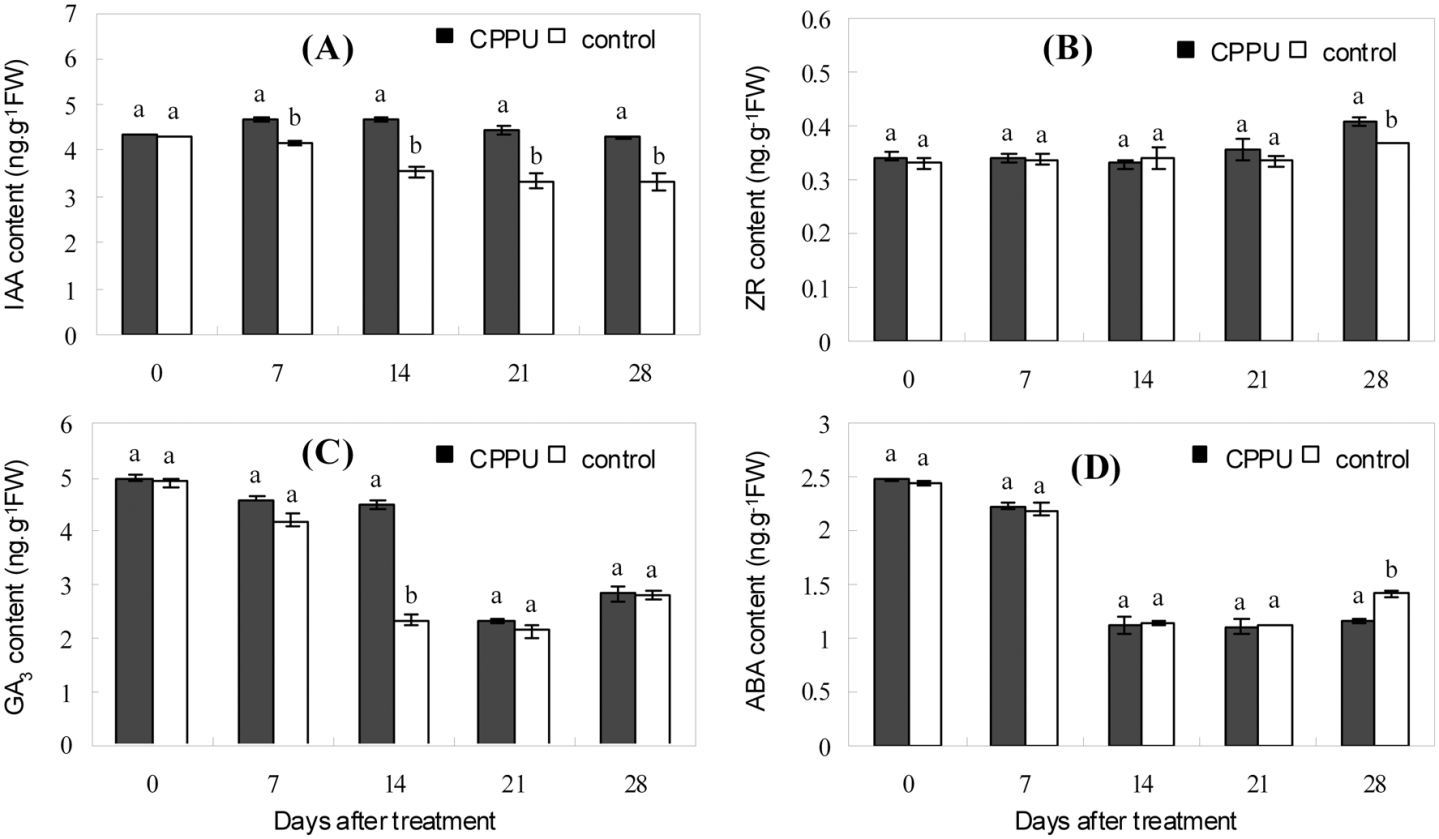
Effect of fruit raceme soaking with CPPU on the endogenous hormones in the husk. Different letters indicate significant difference between the treatment and the control based on *t*-test (*p*<0.05).

**Fig 6:**
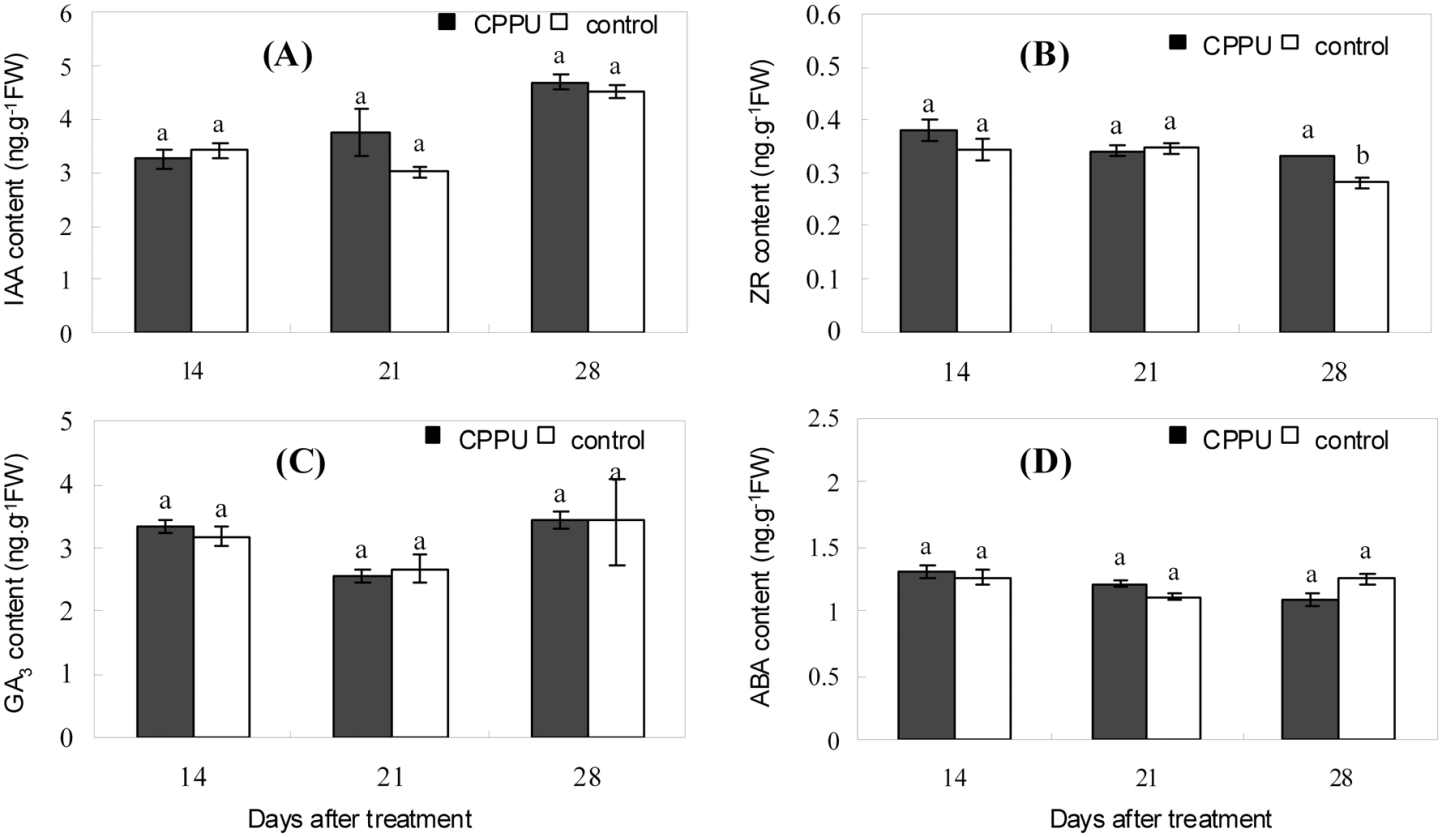
Effect of fruit raceme soaking with CPPU on the endogenous hormones in the seed. Different letters indicate significant difference between the treatment and the control based on *t*-test (*p*<0.05).

**Fig 7:**
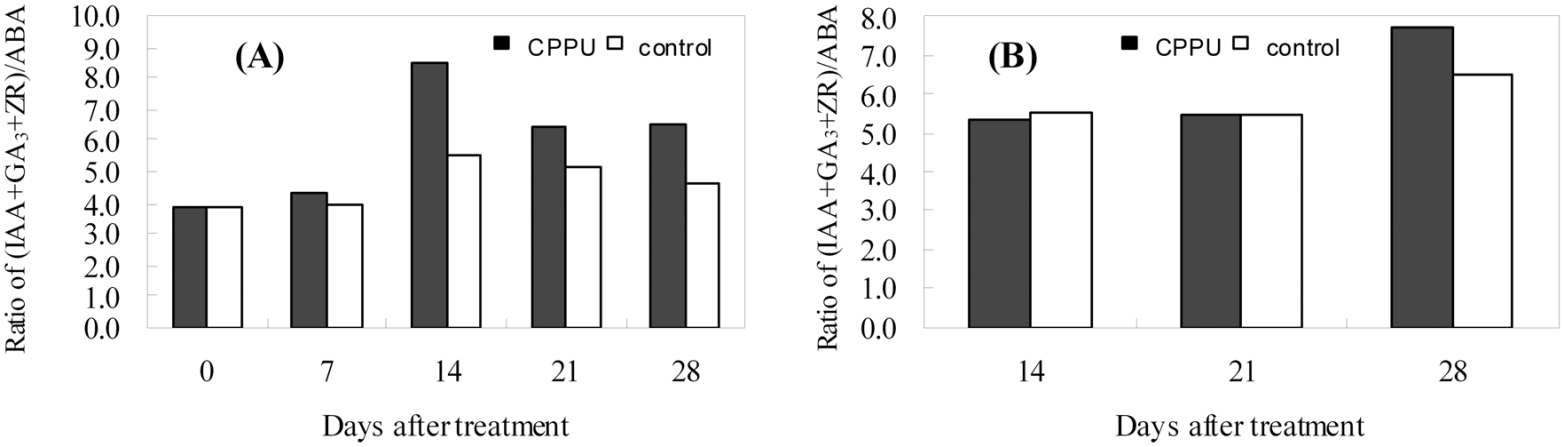
Changes in the ratio (IAA+GA_3_+ZR)/ABA in the husk (A) and seed (B) after CPPU treatment.

The IAA, GA_3_, and ABA levels in the husk appeared to decrease during early fruit development, but the ZR level was relatively constant (Fig 5). In the husk after CPPU soaking treatment, the IAA level was significantly increased (Fig 5A); GA_3_ level was also generally augmented relative to that of the control, and the increase was significant at day 14 (Fig 5C). ZR and ABA levels were not significantly influenced by CPPU treatment until day 28, when ZR was significantly increased while ABA decreased by CPPU (Fig 5C and 5D). Unlike the situation in the husk, CPPU treatment did not significantly influence all the tested hormones in the seed, except for ZR at day 28, which was significantly increased by CPPU treatment (Fig 6). CPPU soaking increased the ratio (IAA + GA_3_ + ZR)/ABA in both the husk and the seed (Fig 7).

## Discussion

Although macadamia trees bear numerous flowers, the excessive abscission of flowers and young fruit is a major problem in macadamia industry. In generally, about 90% of flowers abscise within 2 weeks after anthesis, and 80% of fertilized fruitlets abscise during 3–8 weeks after anthesis [36]. Studies in China showed that the waves of fruit drop occurring within 20–50 days after anthesis accounted for 2/3 to 3/4 of the total abscised immature fruit [9, 10]. Therefore, controlling early fruit abscission is crucial for improving the productivity of macadamia. In the present study, we investigated fruit retention from 15 days after anthesis and confirmed that intensive fruit drop appeared during 15–36 days after anthesis, which led to a fruit drop rate higher than 70%. The results agreed well with those of previous reports [9, 10,36].

A growing body of evidence has revealed that CPPU treatment increased fruit set in many fruit species, such as citrus [21], pear [23], and lychee [37]. In the present study, CPPU treatment significantly reduced young fruit drop within 3 weeks after anthesis, which is similar to the effect of BA treatment during the 21 days after anthesis [2]. Meanwhile, CPPU treatment delayed the peak of fruit drop in macadamia (Fig 1), an effect similar to that of BA applied on racemes before flowering [2]. The influences of foliar spray and raceme soaking with CPPU on accumulative fruit drop were comparable. However, raceme soaking with CPPU was more effective in delaying fruit drop peak than foliar spray.

Fruit is a very strong metabolic sink, and fruit set is tightly related to the availability of carbohydrates [7, 8, 38]. Shortage of carbohydrates resulting in excessive fruit drop has been reported in apple [39, 40], citrus [41], and longan [42]. CPPU has been demonstrated to regulate carbohydrate allocation [43, 44]. After foliar application of CPPU, the levels of starch and total soluble sugars in the leaves were significantly reduced compared with the control, and the reduction in starch occurred earlier than that in soluble sugars, suggesting that CPPU promoted the export of carbohydrates from the source leaves and that the starch was first “consumed” and converted into soluble sugars before being exported. In bearing shoots, both starch and total soluble sugar contents were initially decreased by foliar CPPU application, indicating that CPPU promoted the utilization of carbohydrates in bearing shoots. However, concurrent with the decrease in carbohydrates in the leaves, the contents of starch and total soluble sugars in the bearing shoots were increased by CPPU treatment. Hence, CPPU increased the availability of carbohydrates in the bearing shoots and thus improved fruit retention. The result is similar to that reported in kiwifruit [26] and muskmelon [27].

Fruit raceme soaking with CPPU significantly increased the levels of glucose, fructose, and sucrose in the husk, but did not significantly affect the sugars in the seed. Therefore, CPPU increased fruit retention by increasing the carbohydrate level of the husk rather than the seed, suggesting that the husk might be site where signals for abscission in response to carbohydrate shortage are generated in macadamia. This suggestion agrees with the hypothesis proposed by Botton et al. [40] that abscission signals in response to carbohydrate shortage is initially generated in the cortex tissue of the apple fruit.

Besides carbon nutrition, endogenous hormones are involved in regulating fruit abscission [45]. Raceme soaking with CPPU increased the IAA and GA_3_ levels in the husk and the ZR level in the seed (Figs 5 and 6). The changes in endogenous hormones are favorable for fruit retention, as reported in grape [46], kiwifruit [25], and pomelo [47]. Studies have shown that ABA in fruits could be reduced by CPPU treatment [43, 47]. However, treatment with another CTK-like chemical 6-BA induced starvation stress to apple and thus heavy fruit drop, which was accompanied by an increase in ABA level [48]. ABA may serve as an effector of carbohydrate deficiency to induce fruit abscission [41, 48, 49]. In the present study, CPPU significantly lowered the ABA level in the husk only at 28 days after the treatment and had no significant defect on ABA level in the seed. Therefore, CPPU appeared not to suppress fruit drop in macadamia by reducing ABA. Instead, CPPU modified the balance of endogenous hormones creating a higher ratio of (IAA+GA_3_+ZR)/ABA, which suppressed fruit abscission.

## Conclusion

CPPU can effectively increase fruit retention and delay the occurrence of fruit drop wave in macadamia. During early fruit development, CPPU accelerates the mobilization of carbohydrates from leaves, increases the accumulation of carbohydrates in bearing shoots, and improves carbohydrate availability in fruit. CPPU also modifies the balance of endogenous hormones and increases the ratio of (IAA+GA_3_+ZR)/ABA in the husk chiefly by increasing IAA and GA_3_ levels, which is beneficial for fruit retention. Therefore, CPPU suppressed the early fruit abscission by increasing carbohydrate availability and modifying hormonal balance in macadamia.
